# Electrogastrography in Adults and Children: The Strength, Pitfalls, and Clinical Significance of the Cutaneous Recording of the Gastric Electrical Activity

**DOI:** 10.1155/2013/282757

**Published:** 2013-05-25

**Authors:** Giuseppe Riezzo, Francesco Russo, Flavia Indrio

**Affiliations:** ^1^Laboratory of Nutritional Pathophysiology, I.R.C.C.S. “Saverio de Bellis”, Via Turi 27, I-70013 Castellana Grotte (BA), Italy; ^2^Department of Pediatrics, University of Bari, Policlinico, Piazza Giulio Cesare, I-70124 Bari, Italy

## Abstract

Cutaneous electrogastrography (EGG) is a non-invasive technique to record gastric myoelectrical activity from the abdominal surface. Although the recent rapid increase in the development of electrocardiography, EGG still suffers from several limitations. Currently, computer analysis of EGG provides few reliable parameters, such as frequency and the percentage of normal and altered slow wave activity (bradygastria and tachygastria). New EGG hardware and software, along with an appropriate arrangement of abdominal electrodes, could detect the coupling of the gastric slow wave from the EGG. At present, EGG does not diagnose a specific disease, but it puts in evidence stomach motor dysfunctions in different pathological conditions as gastroparesis and functional dyspepsia. Despite the current pitfalls of EGG, a multitasking diagnostic protocol could involve the EGG and the ^13^C-breath testing for the evaluation of the gastric emptying time—along with validated gastrointestinal questionnaires and biochemical evaluations of the main gastrointestinal peptides—to identify dyspeptic subgroups. The present review tries to report the state of the art about the pathophysiological background of the gastric electrical activity, the recording and processing methodology of the EGG with particular attention to multichannel recording, and the possible clinical application of the EGG in adult and children.

## 1. Introduction

Cutaneous electrogastrography (EGG) is a noninvasive technique to record gastric myoelectrical activity by means of electrodes placed on the abdominal surface. The possibility of using EGG to evaluate normal and abnormal gastric electrical activity, and by inference the gastric motility dysfunction, has great appeal. The interest is largely due to the noninvasiveness and simplicity of this method compared to the established diagnostic techniques, that is, manometry. 

Although a rapid increase in the development of electrocardiography has taken place during the last twenty years, EGG still suffers from several limitation such as difficulties in the recording and analysis of the signal. Some points are still unsettled: which myoelectrical variable can be reliably recorded by EGG? How does EGG correlate with gastric motility and gastric emptying (GE)? And which role does EGG play in the diagnostic workup of patients? 

In the present paper, we will attempt to report the state of the art about the pathophysiological background of the gastric electrical activity, the recording and processing methodology of the EGG with particular attention to multichannel recording, and the possible clinical application of the EGG.

## 2. Gastric Electrical Activity

Gastric electrical activity consists of spontaneous rhythmic electrical activity [1] that determines the timing and frequency of the contractile activity including GE [2–4].

In the stomach, the dominant pacemaker is located along the greater curvature in the corpus and spreads toward the antrum and the pylorus. The concept of “*dominant pacemaker area*” is linked to the highest frequency of slow waves generated in the with corpus respect to other areas of the stomach, allowing that pacemaker area to dominate. In humans, the slow waves frequency in the corpus pacemaker, and so in the entire stomach, is approximately 3 cycles per min (cpm) [5], even if a recent paper has put in evidence several differences with respect to the “standard model” of the gastric electrical activity [6].

Gastric muscles were spontaneously active in each region of the stomach and phasic contractions were associated with slow waves. Morphological studies confirmed that human gastric muscles contain several classes of interstitial cells of Cajal (ICCs): in the myenteric region (ICC-MY) between the circular and longitudinal muscle layers in the corpus and antrum, within muscle bundles (ICC-IM) throughout the stomach, and in septa between muscle bundles (ICC-SEP). ICC-MY are thought to be pacemakers in the stomach because removal of these cells results in loss of slow waves [7]. However, ICC-MY are not the sole pacemakers in most animals, as ICC-SEP/ICC-IM can also generate and propagate electrical activity [8, 9].

These pacemaker currents drive the spontaneous electrical and mechanical activities of smooth muscle cells. The enteric nervous system, composed of both the myenteric (intermuscular) plexus and the submucosal plexus, is also distributed in the gastrointestinal tract from the esophagus to the internal anal sphincter. The role of the ICC and the enteric nervous system in the integrative control of gastrointestinal function and especially of spontaneous rhythmic activity is not well understood. Nevertheless, it is convincing that the ICCs drive spontaneous rhythmic motility, although a role for the enteric nervous system in the regulation of spontaneous rhythmic motility cannot be overlooked. Patterns of contractile activity in gastrointestinal muscles are determined by inputs from enteric motor neurons that innervate smooth muscle cells and interstitial cells [10]. Significant differences between longitudinal and circular muscle layers appear to be related to the amount of cells and contents of ICC-IM. Larger number cells in circular muscle produce dominant electrical signal, and because of tight connections between cells by ICC-IM, simultaneous excitation of circular muscle produces larger electrical signals than that produced by longitudinal muscle.

In ICCs, it is believed that the activation of calcium-activated chloride channels produces a pacemaker potential [11]. The source of Ca for activating the Cl channels is equivocal, and origin from the activity of mitochondria is suggested by some authors [12]. Excitation-contraction coupling occurs by Ca^2+^ entry via ion channels in the plasma membrane, leading to a rise in intracellular Ca^2+^. Ca^2+^ binding to calmodulin activates myosin light chain kinase; subsequent phosphorylation of myosin initiates cross-bridge cycling. Myosin phosphatase dephosphorylates myosin to relax muscles, and a process known as Ca^2+^ sensitization regulates the activity of the phosphatase. In gastric smooth muscle, action potentials may be produced by an opening of voltage-sensitive L-type Ca-channels, while the role of voltage dependent delayed rectifier K-channels may be the termination of membrane depolarization and the maintenance of resting membrane potentials [13].

As in the heart, impairment of pacemaker activity (disruptions in the ICC network or breakdown in the frequency gradient) can induce physical or functional uncoupling of pacemaker activity and affects the efficiency of gastric motility. Uncoupling of pacemaker/ectopic pacemaker can induce alterations in rhythm and rate of gastric electrical activity [14].

## 3. Recording Techniques and Equipment 

The noninvasive recording of the gastric electrical activity by means of cutaneous electrodes was first performed by Alvarez in 1922 on “*a little old woman whose abdominal wall was so thin that her gastric peristalsis was easily visible*” [15]. It was independently discovered again by Davis et al. [16], but the gastric origin of the EGG was conclusively demonstrated only in 1975 by Brown et al. [17].

Usually, EGG has to be recorded in a quiet room to minimize extracutaneous electrical signal which might be detected by the cutaneous electrodes and reduce distractions which could promote patient movement. The patient may be placed in a comfortable position ranging from supine to 45° inclination, and position and comfort should be maintained for the duration of the test. Good quality EGG can be obtained with a sitting subject, enabling the provision of comparable parameters to those achieved from standard examination in a recumbent position [18].

Medications that might modify gastric myoelectrical activity (prokinetics and antiemetics drugs, narcotic agents, anticholinergic drugs, and nonsteroidal antiinflammatory agents) should be stopped at least 1 week prior to testing. 

All components of the EGG recording system must be electrically isolated from the individual to ensure unidirectional current flow from the skin to the amplifiers. All equipments, including the computer, are plugged into a medical grade isolation transformer. 

Usually, pregelled adhesive Ag/AgCl electrocardiographic electrodes are employed, since they allow a reliable acquisition of the EGG signal. Differences in either the size of the conductive area or electrode construction have not revealed a superior choice from among the electrodes, but small dimensions and easy handling may favour the choice of a particular type of EGG examination [19].

Any hair on the abdominal surface has to be shaved to reduce impedance of the skin-electrode interface and an electrode cream is used to improve signal transmission [20]. Both unipolar and bipolar techniques have been employed to record the cutaneous electrogastrogram. The former readily displays the potential variation, whereas the latter gives the best signal to noise ratio and the smallest effect of movement artefacts, respiration, and other signals on the EGG. The bipolar configuration is currently used for EGG recording but the channel number and position depend on the investigators' preferences. So, different configurations of EGG electrodes have been provided with different strength and limitations. Some maps use cutaneous reference points as landmarks for stomach shape and position [21, 22] while alternative set-ups take into account the actual position and shape of the stomach evaluated by means of imaging diagnostics (X-ray or ultrasound) [23, 24] (Table 1).

**Figure 1:**
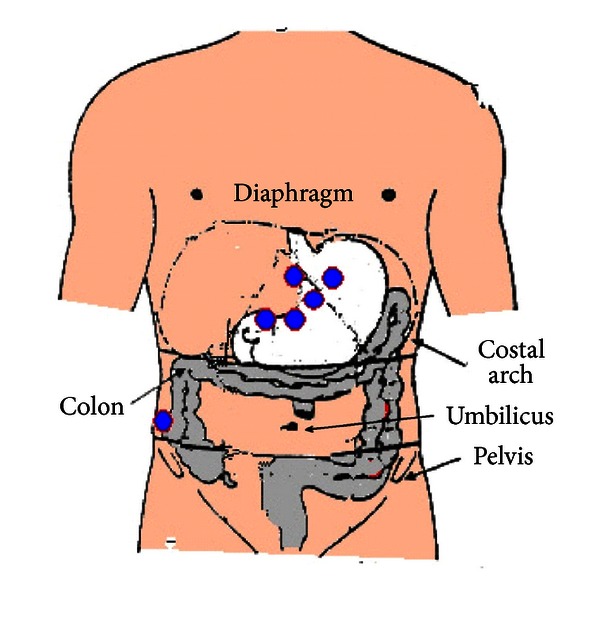
Relationship between viscera and EGG electrodes according to Chen et al. [25].

Recently, Chen et al. [25] used a specific configuration designed to pick up propagation. The electrodes were centred on a main electrode (electrode 3) located 2 cm above the mid-point between the xiphoid process and the umbilicus. Two more electrodes (electrodes 2 and 1) were located on an upper 45 degree angle, with an additional electrode (electrode 4) located 4 cm to the right of the central electrode. The common reference electrode was placed at the cross point of two lines, one horizontal-connecting electrode 1 and the other vertical-connecting electrode 3. The ground electrode was placed on the left costal margin (Figure 1).

After the appropriate skin preparation, usually about 15 min period is needed to establish the electrode-skin interface [26], after which a complete EGG recording consists of a fasting and postprandial recording periods. 

There is no optimal length for an EGG examination; however, a consensus favours the recording of approximately 30 min fasting period followed by a 60 min postprandial period in order to produce reliable and predictable results [27]. Longer ambulatory EGG recordings have been performed, up to 12 hours, but in such cases it is difficult to keep body movement to minimum during the EGG session [28]. 

Riezzo et al. [29] collected evidence for the reproducibility of the EGG signal in 3-day consecutive recordings showing similar frequencies and power. Another study found a good reproducibility in normal subjects but only 91.7% reproducibility for dyspeptic subjects if recordings were performed 1 week apart [30]. In contrast, markedly varied EGG amplitudes were obtained in replicate studies on motion sickness or long lasting EGG recording [31, 32]. 

Motion artefacts still remain a problem in an ambulatory EGG recording and may severely compromise the analysis of the EGG [33]. Measures should be taken to avoid motion artefacts during recordings and to delete them before any kind of analysis. Patients should be advised to remain still and quite whenever possible, particularly during certain period of the recordings (before the meal, the postprandial period, during the onset of a given symptom such as nausea). At present, there is no a reliable method to eliminate motion artefacts. 

The ambulatory recording system has been highly desired for both research and clinical purposes and the development of a small, pocket size EGG recorder realized this issue. The previous ambulatory EGG recorder consisted of a one channel preamplifier, a bandpass analog filter, an analog-to-digital converter, 96 Kb of memory, and a central control unit. It allowed up to 24 h of EGG recording with a sample frequency of 1 Hz. The EGG data could then be stored onto a personal computer and analysed by means of dedicated software. Despite the evident advantage of the pocket EGG recorder, ambulatory EGG recordings worsened the problem of motion artefacts. A further step for the ambulatory EGG has been the development of a multichannel portable system that allows up to 8 bipolar EGG channels. It is well known that a multichannel EGG system is required for the best EGG measurement and for the analysis of the signal propagation [34, 35]. 

## 4. EGG Data Analysis and Interpretation

The cutaneous electrogastrogram is sinusoidal in shape, with a period of about 20 sec and a very weak amplitude, ranging from 50 to 500 micronV, and several signals (i.e., from the small intestine, colon and heart, respiration, and motion) superimposed on it [36]. Noise and the extragastric electrical activity are removed by means of specific internal filters. Adaptive filtering seems to be useful to cancel the respiratory signal [37]. 

Visual analysis is very difficult and time consuming to perform, so real-time digitization and computer analysis have been developed. After recording, the electrogastrogram data are fed into a personal computer and analysed by means of a dedicated software program. Because the EGG signal is approximately sinusoidal in shape, Fast Fourier transformation (FFT) can be applied to the EGG time signal generating a frequency spectrum [38, 39]. 

**Figure 2:**
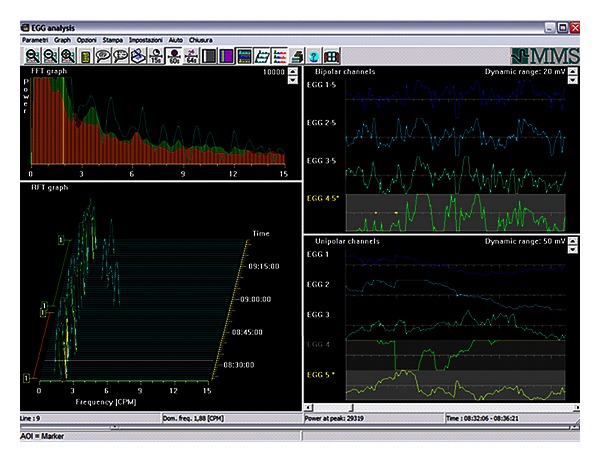
Time signal (bipolar and monopolar time signals, upper and lower, resp.) on the right; Mean Spectrum and Running Spectra Analysis (upper and lower, resp.) on the left. Following Running Spectra Analysis and using Fast-Fourier transform, the frequency components of 256-second (*T*) epochs of EGG signal were calculated, overlapped by 75%  (Δ*T* = 64 s) and displayed as a three-dimensional frequency plot.

An in-deep description of FFT analysis is beyond the scope of the paper; however, the reader can refer to the literature for the peculiar aspect of FFT [20, 40, 41]. In brief, the application of a single FFT to a relatively long EGG signal yields precise information about the main gastric frequency; however, variations in frequency and/or amplitude of the gastric electrical activity cannot be evaluated. To overcome this limitation, van der Schee et al. [42] introduced the running spectral analysis (RSA) making analysis of variations over time in both frequency and amplitude possible (Figure 2).

The clear advantage of spectral analysis is the ability to extract the actual EGG signal from noise; however, the value of the computer analysis should not be overestimated. Visual inspection of both the time signal and frequency spectra is still needed to perform an adequate analysis of EGG signals [43] and so verify the presence of a real episode of tachygastria. The neural networks promise to be affective in eliminating motion artefacts, but it has not been yet currently used in the EGG setting [44]. 

The following parameters are currently accepted and usually calculated for each subject [45].

- Mean dominant frequency (DF) and power (DP) of the EGG are calculated. The frequency/power of the gastric peak is determined by the absolute peak value, and the mean frequency/power is computed by averaging the individual spectra. The normal gastric frequency in healthy humans is approximately 3 cpm ranging from narrow 2.5 to 3.75 cpm or covering a broader 2.0–4.0 range with a slight variation among different centers [20, 40, 41, 45, 46].
- The percentage of the gastric dominant frequency is divided in three ranges defined as normal, bradygastria, and tachygastria (Table 2). A high frequency wave of 4.0–10.0 cpm may be considered tachygastria if there are no normal slow waves, but it may also just be an interference if normal-range gastric slow waves are present at the same time. Signal with a frequency higher than 10 cpm originates from respiration or from electrical activity of other parts of the gut, such as small intestine and colon. In healthy subject, normal slow wave percentage is ≥70% of the recording time [47].
- The power ratio (PR) is used since the absolute values of EGG power are influenced by several factors (skin conductance, distance between the electrodes and the wall of the stomach, variable shapes of the stomach, etc.). Thus, the EGG power can only be evaluated as ratio of postprandial to fasting EGG power values. A PR ≤ 1 is believed to correlate to an impaired GE time [45].
- The instability coefficient (IC) has been introduced to define the characteristic variation in dominant frequency within the normal range. It is calculated as the standard deviation divided by the mean value of frequency: the lower the IC value, the more stable the DF displayed [48].

No information is available regarding rate and direction of propagation. Recently, a multichannel recording device with identical multiamplifiers and an appropriate arrangement of abdominal electrodes seems to be able to detect the coupling of the gastric slow wave from the surface electrogastrogram [34, 35].

## 5. Validation of EGG

Several authors have validated surface EGG by simultaneously recording cutaneous and internal (serosal and mucosal) EGG. In a canine model, Smout et al. [49] found that EGG frequency was the same as that of gastric slow waves recorded by serosal electrodes, and increases in the amplitude of the EGG were associated with the spikes observed in serosal recordings. Hamilton et al. [50] reported similar findings in a human study using simultaneous surface and mucosal electrodes. Pezzolla et al. [51] demonstrated that the electrical activity recorded from the abdominal surface and analyzed by FFT provides reliable information on the actual electrical activity of stomach before and after total gastrectomy. Finally, Familoni et al. [52] performed simultaneous surface and serosal recordings in postoperative patients reporting that normal slow waves as well as dysrhythmias can be detected by EGG. 

In a 24-hour ambulatory EGG performed in healthy volunteers, a stable normogastric rhythm was omnipresent, and EGG frequency and amplitude increased after meals. The mean gastric frequency showed a clear circadian variation and the dominant frequency was lower during the night than in the day. Gastric frequency was associated with short periods of tachygastria in some of the volunteers but none of the tachygastria periods was associated with symptoms. Variations in amplitude should be due to gastric activity and/or changes in the position of the stomach [53, 54], and decreased amplitude may depend on the propagation rate of depolarisation [55], or it can be caused by uncoupling of electrical control activity [56]. Artefacts were very common but there was no difference in signal yield from different time of the day or the night [57].

### 5.1. Patients Characteristics

Patient characteristics are reported to affect the EGG signal, among them age, ethnicity, and body mass index (BMI). As regards age, in elderly groups the ratio of the incidence of the 3 cpm wave during the postprandial period compared to the fasting state was reduced compared to young subjects, and the reduction was greater in the inactive elderly than in the active elderly group. The PR was also lower in the elderly groups. The GE time was delayed in the active elderly and more so in the inactive elderly group [58]. Multichannel EGG provides assessment of electrical slow-wave coupling in addition to determining dominant frequency, power, and percentage normal rhythm. A multicenter study of normal subjects shows similar multichannel EGG values among different genders and ages. Body mass and ethnicity may impact on some of the EGG values [59].

The normal values of electrical activity in children and the effects of age, gender, and BMI are not well defined. To evaluate these items, EGG signal was picked up before and after a meal in 114 healthy children by means of a pair of cutaneous electrodes sonographically placed on the abdominal surface. The dominant frequency was prevalently found in the 2.0–4.0 cpm range. A significant difference was found comparing the pre- and postprandial instability coefficient of dominant frequency. The EGG power increased postprandially, and the PR was not correlated to the approach of the wall of the gastric antrum to the abdominal surface. In children 6–12 years old, EGG parameters are not affected by BMI in response to a mixed test meal. Gastric electrical rhythm and rate and gastric power are not influenced by age and gender, whereas the instability coefficient seems to be influenced by these factors. The normal values of the EGG parameters evaluated in this study should be introduced in the analysis of gastric electrical activity for an effective interpretation of the EGG signal from children with functional or organic diseases [60].

### 5.2. Food Intake

Food intake is assumed to be a physiological stimulus capable of activate or modulate the hormonal and mechanical activity of the stomach. The meal ingestion induces an increase in the EGG amplitude [61] that runs parallel with gastric electrical response, gastric distension, and antral contractions [62] and is influenced by the physical and chemical characteristics of the meal. Using a muffin-based test meal, the postprandial to fasting PR for a 350 kcal meal was greater than that for a 250 kcal meal and the half empting time for the 350 kcal meal was significantly longer than that for a 250 kcal meal [63]. 

Considering the association between postprandial power and the nutritional composition of the test meal, some authors investigated the effect of fat preload on gastric myoelectrical activity in normal humans and showed that a fat preload decreased the power of the EGG significantly but does not affect the frequency of the gastric slow waves [64].

Comparing the effects of the osmolarity of different drinks, the isotonic glucose drink elicited a positive chronotropic influence on and stabilization of EGG with respect to distilled water. At the transition from isotonicity to hypertonicity, a pronounced bradygastria was recorded after the energy-free hypertonic NaCl solution, whereas a hypertonic glucose solution evoked a tachygastric pattern. A marked delay in GE was found with both hypertonic drinks. Of the five fluids examined, isotonic glucose appeared to be the drink least disturbing to EGG, so osmolality and its interplay with chemical composition/energy density must be taken into account when choosing a test meal for an EGG examination [65]. The viscosity of food can affect gastric motility and gastric empting. In healthy individuals, pectin increased the viscosity of enteral nutrition and accelerated GE [66] whilst the addition of guar gum to a semisolid meal up to a dosage of 4.5 g did not affect GE and intestinal transit [67]. 

The effect of enhanced viscosity introducing meals with three different percentage of galactomannan was tested in dogs showing a delayed GE, an increased postprandial intestinal but not gastric motility, and no effects on canine gastric and intestinal slow waves [68].

### 5.3. Pressure Activity

To evaluate the reliability of the EGG, EGG parameters have been compared to parameters obtained from other techniques measuring the gastrointestinal motility. The relationship between EGG signal and pressure activity was assessed by a fluoroscopic study showing a 3 cpm frequency corresponding to 3-cpm gastric contraction while higher or lower frequencies could cause abnormal gastric peristalsis [69]. A one-to-one association of the EGG and antral contractions was shown during fluoroscopy only in the case of antral contractions of high amplitude, while low amplitude contractions failed to affect the EGG amplitude. On the contrary, simultaneous recordings of EGG signal and sonographically measured antral contraction did not reveal any correlation between the amplitude of EGG signal and the mechanical activity of the stomach [70]. A more recent paper on the same topic confirmed that EGG and antroduodenal manometry measure different aspects of gastric motor activity and cannot show a spatial correlation [71]. On the same matter Sha et al. [72] confirmed that no one-to-one correlation was noted between the symptom score and any of the EGG or motility parameters. EGG and antroduodenal manometry can complement each other in demonstrating gastric motor dysfunction in patients with functional dyspepsia.

### 5.4. Gastric Emptying

Controversial findings have been reported in the literature regarding the correlation between EGG and GE. Koch et al. [73] have shown no association between EGG and GE, whilst Abell et al. [74] have found a good positive correlation between dysrhythmic EGG and delayed GE. A positive correlation between postprandial EGG amplitude and the degree of gastric antral area has also been found by des Varannes et al. and Chiloiro et al. in primates and humans, respectively [75, 76]. However, there is no firm evidence of a functional correlation between a normal EGG and a normal GE. A normal EGG recording may not guarantee a normal GE, but an abnormal EGG may predict a delayed GE. Nearly 50% of patients with dyspepsia had an impaired GE [77]. Patients with both delayed GE time and abnormal EGG had more severe symptoms and more tachygastria in pre- and postprandial states than patients with normal GE [78]. 

Overall, the positive predictive value of an abnormal EGG to predict gastroparesis ranges from 50% to 81% (average 65%), whereas the accuracy of a normal EGG to predict normal GE in a symptomatic population ranges from 65% to100% (average 76%) [77, 79–81].

The use of the multichannel EGG equipment to correlate EGG and GE time parameters has confirmed the correlation between the two procedures, namely, the temporal regularity and spatial regularity of gastric slow waves negatively correlated with GE time. This suggests that the impaired gastric myoelectrical activity expressed as uncoupling might be responsible for the delayed GE in patients with functional dyspepsia [82].

### 5.5. Drugs

EGG has been shown to be of potential clinical relevance as objective measure of the effects of putative therapeutic agents in selected patients. Regarding prokinetic agents, domperidone [73] and erythromycin [83] seem to induce a normal gastric myoelectrical activity together with an improvement in upper gastrointestinal symptoms and emptying time. However, their ameliorating effects on symptoms of dyspepsia are often limited. The major reason is that the prokinetics do not improve or even impair gastric accommodation that is one of the major pathogenesis of gastroparesis and functional dyspepsia [84]. Trimebutine maleate, another prokinetic drug, when associated to a proton pump inhibitor may improve gastric motility in patients with gastric ulcer by inducing a clear reduction in periods of tachygastria and bradygastria periods but with no effects on GE [85].

Cisapride improved gastric dysrhythmia in patients with diabetic gastroparesis. In fact, upper gastrointestinal symptoms and GE of indigestible solid showed significant improvement after 8 weeks of cisapride treatment [86]. Cisapride is no more in the market because of ECG side effects and mosapride has been tested in nonerosive gastroesophageal reflux (NERD) showing an improved gastric electrical activity and emptying compared with pretreatment values [87]. Prokinetic drug treatments (itopride hypochloride, mosapride, and levosulpiride) are useful in improving dyspepsia symptoms via an improved gastric electrical activity [88]. 

Octreotide, a somatostatin analogue, exerts an inhibitory effect on postprandial myoelectrical activity and gastric motility suggesting caution in the use of octreotide in patients with gastroparesis [89].

A study on the effects of Proton Pump Inhibitor, omeprazole, on gastric electrical activity and GE did not show any significant difference in the half emptying time but improved gastric myoelectrical activity. These effects of omeprazole may be one of the mechanisms involved in its efficacy in relieving dyspeptic symptoms in patients with functional dyspepsia [90].

## 6. Clinical Significance of EGG

EGG is not able to diagnose a specific disease, but it provides evidence of stomach motor dysfunctions in parallel to other techniques such as manometry and GE studies. Disorders in the gastric electrical rhythm and rate [91, 92] may be a cause of gastric dysmotility [93, 94]. In particular, dysrhythmias have been found in association with gastroparesis [81], namely, bradygastria and bradyarrhythmia [95]. Chen and McCallum have classified EGG abnormalities in patients with gastroparesis into the following categories: pre- and/or postprandial dysrhythmias and decreased EGG amplitude after a test meal. These abnormalities were significantly more common in gastroparetic patients than normal subjects [47]. 

Gastric myoelectrical pattern recorded in the EGG can distinguish between mechanical and idiopathic causes of gastroparesis. Patients with gastroparesis due to outlet obstruction had high amplitude and excessively regular 3 cpm activity, whereas patients with idiopathic gastroparesis had primary 1 to 2 cpm activity and poor 3 cpm activity [96]. In 80% of patients, delayed GE time and tachygastria recorded by means of EGG correlate positively [97]. Patients with gastroparesis and abnormalities of EGG rhythm and power had delayed GE. Patients with delayed GE have a lower percentage of normal gastric slow waves in the EGG and a lower postprandial increase in the dominant power. Abnormalities in the postprandial EGG seem able to predict delayed GE but a normal EGG does not guarantee a normal emptying time [77]. 

Nausea and abdominal pain are the most common complaints of patients with gastroparesis. Some patients with diabetic gastropathy exhibit cyclical symptoms such as cycling vomiting alone or associated with migraine headaches, and delayed GE. These patients may present more abnormal EGG frequencies than other patients with diabetic gastroparesis but no cyclical symptoms [98]. 

A normalization or improvement in gastric electrical activity, associated with significant acceleration of GE, was found after treatment with prokinetics [99]. In the case of gastroparesis refractory to standard medical therapy, gastric pacing seems to be effective to improve symptoms and to accelerate GE time [100, 101].

EGG may be a very attractive method to investigate the pathophysiology of nausea and vomiting in pregnant women [102]. Koch et al. [103] have performed EGG in pregnant women with and without nausea. Gastric dysrhythmias were observed in the first group, while normal slow waves were seen in the second group. In a similar study, Riezzo et al. [48] have found EGG abnormalities in symptomatic patients that disappeared after voluntary interruption of pregnancy. Progesterone induced an increase in power of the bradygastria signal and the simultaneous administration of estradiol and progesterone in doses reproducing pregnancy levels induced an additive effect with increased bradygastria and tachygastria in nonpregnant women [104]. In pregnant women, meals with a predominant protein content reduced nausea and dysrhythmic activity more than carbohydrate and fat meals with identical caloric content, and liquid meal decreased dysrhythmias more than solid meals [105]. 

In many patients with dyspeptic symptoms no organic cause can be found, so that the term nonulcer or functional dyspepsia is applied. About half of these patients have a delayed GE and further investigations, such as manometry and EGG, often disclose antral hypomotility and a disordered electrical activity with respect to patients with normal GE [25]. The EGG was found to be abnormal from 36% to 60% of functional dyspeptic patients [79] with an ability to detect a dysfunction of gastric motility of approximately 93%, so EGG is able to define a subgroup of patients with functional dyspepsia and electrical rhythm disturbances. Tachygastria is associated with absence of antral motility in animal model [106] and increased plasma vasopressin, epinephrine, and nausea scores [107]. In the case of an arrhythmogenic gastric focus, its removal led to clinical improvement [99]. In irritable bowel syndrome (IBS), the electrogastrogram is abnormal only if concurrent dyspepsia is present [108]. Including the percentage of slow wave coupling, both the temporal regularity and spatial regularity of gastric slow waves have negative correlations with GE, which suggests that the uncoupling may be responsible for the delayed GE in patients with functional dyspepsia [82]. 

Gastric colonization with *H. pylori* (*Hp*) did not seem to affect EGG and GE parameters [78], but some authors have found increased percentage of tachygastria and antral hypomotility [109] as evidenced by the absence of phase III of MMC in *Hp* positive patients. Dyspeptic patients had a higher dominant power, a lower percentage of normal 3 cpm activity,and a higher percentage of tachygastria compared to healthy control subjects [110]. When *Hp* status was considered, these significant differences persisted in both *Hp *positive and negative dyspeptic patients compared to *Hp *negative controls. However, the instability coefficient of the dominant power, representing the variability of the EGG amplitude within the normal slow wave range, was very high in *Hp-*positive controls and this could be considered as an early index of *Hp* infection. 

Several studies have investigated the role of gastric peptides in patients with dyspepsia and IBS [111–113]. In particular, considering the *Hp*-associated dyspepsia, gastrin levels increase during *Hp* infection and return to normal after eradication of the infection [114]. *Hp *positive asymptomatic subjects showed higher gastrin level than *Hp *negative ones, suggesting that hypergastrinemia in the early phase of *Hp* infection predisposes or contributes to *Hp*-related dyspepsia [110]. 

Stern's EGG studies on motion sickness have provided new insights concerning the correlation between dysrhythmias and nausea [115]. In these studies, a rotating optometric drum serves as a provocative stimulus to create illusory self-motion and the sensory conflict provokes motion sickness in approximately 50% of healthy subjects. It seems that subjects who experience nausea in the rotating period show an initial increase in sympathetic nervous system activity and a decrease in parasympathetic activity, followed by a change in gastric electrical activity from normal to tachyarrhythmia. Gastric dysrhythmia precedes the onset of nausea and the increase in vasopressin release. Gastric dysrhythmias can alter vagal afferent nerves and they, in turn, may directly induce a sensation of nausea or increase levels of vasopressin [107]. The latter may be experienced by patients as nausea.

As regards organic diseases, the characteristics of myoelectrical rhythm in gastric cancer (GC) patients were reported, indicating that advanced GC was the factor responsible for the obvious dominant power enhancement after meal, whilst demographic, clinical, and other cancerous factors did not influence EGG parameters [116, 117]. The disturbance of gastric electrical control activity in patients with chronic idiopathic intestinal pseudoobstruction (CIIP) consists of dysrhythmia and discriminates between primary pathologies (neural or muscular ones) [118, 119].

Diabetic gastropathy is a disorder that occurs in both type 1 and type 2 diabetes. Patients often present with nausea, vomiting, bloating, early satiety, and abdominal pain of different levels of severity. The pathogenesis of this complex disorder is still not well understood but involves abnormalities in multiple interacting cell types including the extrinsic nervous system, enteric nervous system, ICCs, smooth muscles, and immune cells. The primary diagnostic test remains gastric scintigraphy, although other modalities such as breath test, capsule, ultrasound, magnetic resonance imaging (MRI), and single photon emission computed tomography (CT) imaging, show promise as alternative GE diagnostic modalities [120]. The mainstay of treatment for diabetic gastroparesis has been antiemetics, prokinetics, nutritional support, and pain control. In recent years, on the basis of several studies on the alteration of gastric electrical activity in diabetic gastroparesis, gastric stimulation has been used in refractory cases with nausea and vomiting [121]. 

The studies on gastrointestinal motility in neonates concern the ontogeny of the electrical and mechanical activity of the gut [122]. The EGG studies have demonstrated that gastric electrical signals are similar in preterm and term newborns [123–125]. Koch et al. [123] found no difference between preterm and full-term infants, but another study found the gastric electrical activity to be immature at birth suggesting a development process from 1 week to 6 months [26]. In another study on ontogeny in preterms performed by means of EGG and ultrasonography, a clear maturation pattern of gastric motility was shown [126]. An abnormal EGG pattern and delayed GE time were present in preterm infants of 28–32 weeks of age. The 3 cpm activity became dominant at approximately 32 to 36 weeks of gestational age, and by that time, the GE time was similar to that of full-term infants. Although enteral feeding is important for the development of gastrointestinal motility, gastric electrical activity and emptying show an intrinsic maturation pattern depending on the gestational age. This datum has been recently confirmed by Chen in a paper on the development of gastric slow waves and effects of feeding in preterm and full-term infants [127]. 

The gastric electrical activity and emptying were studied along with the intestinal permeability in preterm newborns to verify if a maturation of the mucosal barrier exists in preterm newborns during the first month of life. Both electrical and motor activities are completely developed at birth, whilst the intestinal epithelial barrier evaluated by lactulose/mannitol test clearly improves during the first week of life [128]. 

EGG can be used in the diagnostic workup of children with dyspepsia, where the presence of gastric dysrhythmia strongly suggests a functional origin of symptoms [129]. Pre-and postprandial gastric electrical abnormalities were found in a high proportion of pediatrics patients with dyspepsia. Children with functional dyspepsia have a wider frequency range, a higher percentage of tachygastria and a reduced postprandial amplitude increase if signal is compared to healthy children [130, 131]. These changes were associated with delayed GE and a delayed GE was present in 66% of the dyspeptic children. 

Vomiting is common in children with disorders of the central nervous system (CNS) and it is commonly attributed to gastroesophageal reflux. Among children with CNS disorders and vomiting, gastric dysrhythmia was present in 62% of the patients and 32% of patients had gastroesophageal reflux and gastric dysrhythmias [132]. 

Abnormal gastric myoelectrical activity may play a role in the pathogenesis of cyclic vomiting syndrome (CVS) [133]. Children with CVS have a characteristic periodicity, and this could be due to abnormal gastric myoelectrical activity detectable by EGG. CVS children showed marked episodes of tachygastria preprandially and all showed tachygastria postprandially. The asymptomatic CVS children showed tachygastria only postprandially after the test meal. Abnormal EGGs and higher tachygastria activity or PRs were associated with delayed GE. 

Chronic renal failure showed gastric antral dysrhythmias, such as bradyarrhythmia and tachyarrhythmia, and a close relationship between nausea and gastric electrical abnormalities. After renal transplantation gastric dysrhythmias disappeared [134, 135]. Unlike adults [136], children with anorexia nervosa did not show EGG abnormalities [137]. 

CIIP is usually a primary disorder in children, but it is often secondary to diabetes or sclerodermia in adults. It is an uncommon disorder of intestinal neural or muscular function characterized by recurrent episodes of intestinal obstruction without an evident anatomical cause, vomiting, and intolerance of enteral feeding. EGG has been proposed as first-line approach in children with CIIP. In fact, in these patients, a persistent tachygastria seems to be highly suggestive of a neuropathic dysmotility, while in cases with no dominant frequency the motor disorder in the gut is usually myopathic [138]. 

The gastric electrical activity in healthy full-term newborns fed with formula or breast-milk was compared during the first 6 months of life [139]. An adult-like gastric 3 cpm activity can be observed in breast-fed newborns in contrast to formula-fed ones, probably as an effect of colostrum. Infant formulas containing hydrolyzed cow milk protein are usually administered to reduce feeding intolerance and to improve GE. However, no differences were found in terms of gastric electrical activity and GE time between the two groups, standard formula, or hydrolyzed formula so it seems unnecessary to use hydrolysate formulas to improve motility in preterm infants [140]. 

In sensitized infants, cow's milk induces severe gastric dysrhythmia and delayed GE, which in turn may exacerbate GE and induce reflex vomiting. EGG and the evaluation of GE can be useful in the assessment of vomiting, gastroesophageal reflux, and cow's milk allergy in infants [141]. 

An important field of interest is represented by the use of probiotic and prebiotic enriched formulas to improve feeding tolerance. Indrio et al. [142] demonstrated that probiotic enriched formula did not induce differences in the EGG parameters with respect to breast-milk and standard formula but a smaller fasting antral area was found in preterms fed with formula added with probiotics and GE rate was significantly faster as well. All together these findings could be markers of a reduced gastric residual in newborns fed with probiotics respect to formula fed newborns. The clinical counterpart of this physiological condition may be the reduced numbers of regurgitation as reported in the previous paragraph [141]. Safety and tolerance of a probiotic formula with Lactobacillus reuteri was recently described in full-term infants [143]. 

Prebiotics are soluble fiber able to modify the colonic microflora and are preferred with respect to probiotic in the neonatal feeding, particularly for preterm newborns. In a double-blind, randomized, placebo-controlled study was evaluated the effect of a prebiotic mixture of oligosaccharides on gastric motility in preterm newborns showing that prebiotic may improve the tolerance of enteral feeding. In fact, the percentage of time in which propagation was detected in the electrogastrography signal was twice in newborns receiving formula with prebiotics respect to placebo, and the gastric half-emptying time was 30% faster in the prebiotic group than the placebo group infants [144].

## 7. Future of EGG

It is evident that many issues must be addressed in order to make the EGG a reliable diagnostic test for patients with gastric motility dysfunction. Until now, there has been no standard recommendation for electrode position, length of EGG recording, and test meal as well the EGG systems and analysis. Besides, defined normal ranges are discrepant among many investigators. It appears that an acceptable consensus is needed to define the abnormalities based on the EGG recording when it is used to assess stomach motor dysfunction. The central question whether dysrhythmias are causative of a particular gastrointestinal syndrome or if they are epiphenomena still remains an open question. 

An ambulatory multichannel EGG recording system is now available for long-lasting recordings of normal and impaired gastric electrical activity along with a specific software to look behind the only EGG frequency analysis. The next generation of EGG systems has to integrate an advanced analysis allowing to provide information in terms of slow wave coupling/uncoupling rather than episodes of dysrhythmia and to define and mapping the ectopic pacemakers responsible for dysrhythmias. Neural network to automatically delete motion artifacts may be an important step in the development of ambulatory EGG. 

To partly overcome the current pitfalls of EGG, a multitasking diagnostic protocol could involve the EGG recording and the evaluation of the GE time, along with validated gastrointestinal questionnaires and biochemical evaluations of the main gastrointestinal peptides. 

Different studies have demonstrated that ^13^C-breath test is a reliable method to measure solid phase emptying. The results are reproducible and correlate with scintigraphic GE results [145]. Besides, MRI has developed as another noninvasive method for detailed evaluation of GE, motility, and volume without the disadvantages of ionizing radiation and without the use of intragastric catheters that influence gastric physiology [146, 147]. Several peptides have been demonstrated to be involved in the onset and/or modulation of the dysrhythmias in several experimental models. A comprehensive evaluation of all these variables could identify different subsets of dyspeptic patients in a noninvasive way.

In conclusion, solving all the procedural methods, EGG may be an important step in the definition of patients with peculiar gastrointestinal diseases and the followup of patients involved in pharmacological treatment and intervention diets.

## Figures and Tables

**Table 1:** Some maps of electrodes in the EGG recording.

Authors	Stomach position	Electrode position	Coordinates	Notes
Mintchev et al. [21]	Cutaneous reference points	Proximal electrode	5 cm to the left of the xiphoid process on the costal margin	Commonly used
		A row of 4 electrodes (3 cm apart)	Between the first electrode and the junction of the mid-clavicular line with the right costal margin	

Patterson et al. [22]	Cutaneous reference points	One electrodes	Between the umbilicus and the xyphoid process	Alternative ones
		Second electrode	Left side of the abdomen, one-third of the distance from the ventral mid-line to the left axial mid-line, 1 cm below the lowest rib	

Koch and Stern [23]	Sonography	2 electrodes	Surface overlying the antropyloric axis calculated by ultrasonography	Best signal to noise ratio
		The reference electrode	Placed to form an equilateral triangle	

Mirizzi and Scafoglieri [24]	X-Ray/sonography	A daisy-chain of 6 electrodes (3 bipolar channels)	Surface overlying the antro-pyloric axis calculated by ultrasonography/X-ray for at least one channel	Best signal to noise ratio

Chen et al. [25]	Cutaneous reference points	Five electrodes	The main electrode located 2 cm above the mid-point between the xiphoid process and the umbilicus. Three more electrodes were located on an upper 45 deg angle, with an additional electrode located 4 cm to the right of the central electrode. Reference electrode on the right lower quadrant	Designed to pick-up propagation

**Table 2:** Definitions of spectral EGG frequencies.

Normal	Peaks in frequencies ranging from 2.0 to 4.0 cpm
Normal with harmonics	Peaks in frequencies ranging from 2.0 to 4.0 cpm with peaks at exact multiples of the fundamental frequency
Bradygastria	Peaks in frequencies ranging from 1.0 to 2.0 cpm in absence of a peak in the normal range
Tachygastria	Peaks in frequencies ranging from 4.0 to 10.0 cpm in absence of a peak in the normal range
Noise/no signal	No clear peaks, or multiple peaks in the spectrum, strong elevation of the baseline, significant noise or artefacts in the row EGG signal
